# Does Time Really Slow Down during a Frightening Event?

**DOI:** 10.1371/journal.pone.0001295

**Published:** 2007-12-12

**Authors:** Chess Stetson, Matthew P. Fiesta, David M. Eagleman

**Affiliations:** 1 California Institute of Technology, Pasadena, California, United States of America; 2 Department of Neurobiology and Anatomy, The University of Texas Medical School at Houston, Houston, Texas, United States of America; 3 Department of Neuroscience, Baylor College of Medicine, Houston, Texas, United States of America; 4 Department of Psychiatry, Baylor College of Medicine, Houston, Texas, United States of America; Istituto di Neurofisiologia, Italy

## Abstract

Observers commonly report that time seems to have moved in slow motion during a life-threatening event. It is unknown whether this is a function of increased time resolution during the event, or instead an illusion of remembering an emotionally salient event. Using a hand-held device to measure speed of visual perception, participants experienced free fall for 31 m before landing safely in a net. We found no evidence of increased temporal resolution, in apparent conflict with the fact that participants retrospectively estimated their own fall to last 36% longer than others' falls. The duration dilation during a frightening event, and the lack of concomitant increase in temporal resolution, indicate that subjective time is not a single entity that speeds or slows, but instead is composed of separable subcomponents. Our findings suggest that time-slowing is a function of recollection, not perception: a richer encoding of memory may cause a salient event to appear, retrospectively, as though it lasted longer.

## Introduction

Temporal judgments – such as duration, order, and simultaneity – are subject to distortions [1]. For example, perceived durations can be warped by saccades [2], [3] or by an oddball in a sequence [4]. Temporal order judgments of actions and sensations can be illusorily reversed by exposure to delayed motor consequences [5], and simultaneity judgments can be manipulated by repeated exposure to non-simultaneous stimuli [6]. Distortions in interval timing can be induced by narcotics such as cocaine and marijuana [7], as well as by disorders such as schizophrenia [8] and Parkinson's disease [9].

However, an open question is whether subjective time is a unitary phenomenon, or instead whether it is underpinned by separate neural mechanisms that usually work in concert but can be dissociated under the right circumstances. In other words, when one temporal judgment changes, do the others necessarily follow suit?

To address this question, we turned to the common anecdotal report that time seems to have slowed down during a life-threatening situation (such as a car accident). The experimental question, for the present purposes, is what it might mean for ‘time’ to move in slow motion. If time as a single unified entity can slow down – the way it does in movies – then this slow motion should entail consequences such as an ability to perceive events with higher temporal resolution. For example, watching a hummingbird in video slow motion allows finer temporal discrimination upon replay at normal speed because more snapshots are taken of the rapidly beating wings. To test whether humans experience increased temporal resolution during frightening events, we designed an experiment in which participants could accurately detect a visual stimulus only if they were experiencing supra-normal temporal resolution.

## Methods

We leveraged the fact that the visual brain integrates over a small window of time. If two or more stimuli arrive within a single window of integration (usually ∼80 msec), they are perceived as a single stimulus [10]. As an illustration of this principle, a child's toy known as a thaumatrope has, for example, a picture of a bird on one side of a disc and a picture of a tree branch on the other; when the disc is wound up and spun quickly so that both sides are seen in rapid alternation, the bird appears to be resting on the branch. Because the stimuli are alternating so rapidly that the visual system cannot distinguish them temporally; they are seen as though simultaneously present.

**Figure 1:**
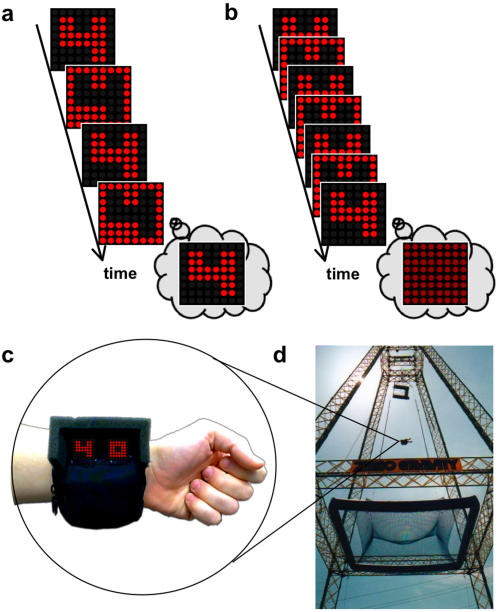
Measuring temporal resolution during a fearful event. (a) When a digit is alternated slowly with its negative image, it is easy to identify. (b) As the rate of alternation speeds, the patterns fuse into a uniform field, indistinguishable from any other digit and its negative. (c) The perceptual chronometer is engineered to display digits defined by rapidly alternating LED lights on two 8×8 arrays. The internal microprocessor randomizes the digits and can display them adjustably from 1–166 Hz. (d) The Suspended Catch Air Device (SCAD) diving tower at the Zero Gravity amusement park in Dallas, Texas (www.gojump.com). Participants are released from the apex of the tower and fall backward for 31 m before landing safely in a net below.

We applied this characteristic of the visual system to the perception of rapidly alternating digits. When shown a digit and its negative image at a slow rate of alternation (Fig. 1a), participants have no trouble identifying it. As the rate of alternation increases, a sharp threshold is reached after which the information is presented too rapidly and the digit can no longer be discriminated from a uniform display (Fig. 1b) – that is, at a high rate of alternation the images perceptually overlap as though they were presented simultaneously.

Using this technique of rapid alternation, we were able to measure a participant's temporal threshold both before and during a frightening event to determine if there was any change in resolution. We engineered a portable device (the “perceptual chronometer”, Fig. 1c) which contains 2 LED arrays and a programmable microchip that presents randomized digital numbers and their negatives at adjustable rates. Participants watched a briefly presented (∼2 sec), rapidly alternating pair of digits on the perceptual chronometer, and, for different alternation frequencies, attempted to verbally report the digits they perceived. The temporal resolution of the viewer determined whether the digits were identifiable or not.

To establish temporal thresholds, each participant was asked to report the numbers shown on the device, starting with a slow repetition frequency. Upon correct digit identification, we randomized the digits and increased the alternation frequency by decreasing the period 6 ms. The threshold was identified as the frequency at which a participant was unable to correctly identify randomized numbers after 3 consecutive presentations. We tested 13 participants during the daytime and 7 at nighttime and found average threshold periods of 47.4±13 ms and 33.4±9 ms, respectively.

After obtaining thresholds, we harnessed participants safely to a platform which was then winched 46 m off the ground to the apex of a Suspended Catch Air Device (SCAD) tower (Fig. 1d). The chronometer was strapped to the participant's forearm like a wristwatch, and was programmed to display a random number alternating at a period 6 ms faster than their determined threshold. Participants were released from the top of the tower and experienced free fall for 2.49 s before landing safely in a net. During the fall, they attempted to read the digits for subsequent report. If higher temporal resolution were experienced during the freefall, the alternation rate should appear slowed, thus allowing for the accurate reporting of numbers that would otherwise be unreadable.

One of the two experimenters remained on the platform to monitor for eye closure. One participant who closed her eyes for the entire free fall was excluded from the study; all others kept their eyes open during at least part of the freefall.

Upon landing in the net, participants verbally reported the displayed number; if they were unsure, they were asked to deliver their best guess. The displayed number was verified by the experimenters by slowing the display to an easily readable alternation rate, and answers were scored for accuracy (correct reporting of both digits = 100%, one digit = 50%, neither = 0%).

Additionally, 7 participants made duration judgments. After watching another participant take the freefall, but before they took the freefall themselves, we asked these participants to imagine being released from the top and then falling through the air until they hit the net. At the moment when they pictured their release from the top, they were to press the start button on a stopwatch; at the time that they imagined hitting the net, they were to press the stop button. We repeated the same measurement just after they experienced the free fall themselves, this time asking them to remember being released from the top and estimate how long it took until they hit the net.

To avoid incentivization, participants were not compensated except for the cost of the SCAD dive. All participants gave written informed consent as approved by the IRB at the University of Texas, Houston Medical School.

## Results

**Figure 2:**
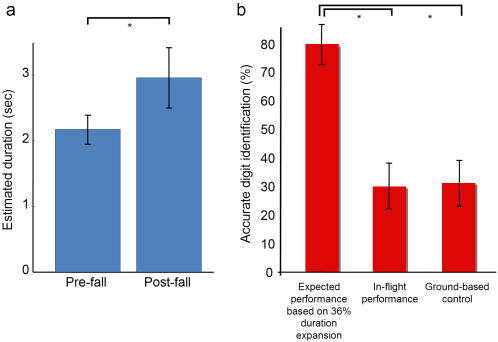
No evidence for fear-induced increase in temporal resolution. (a) Participants' estimates of the duration of the free-fall were expanded by 36%. The actual duration of the fall was 2.49 sec. (b) If a duration expansion of 36% caused a corresponding increase in temporal resolution, a 79% accuracy in digit identification during the fall would be predicted (left bar, see text). However, participants' accuracy in-flight was significantly less than expected based on this theory (middle bar, p<2×10^−6^). In-flight performance was no better than ground-based controls (right bar, p = 0.86), in which the experimental sequence was identical except that the participants did not perform the free fall. The performance scores are averaged over participants, each of whom performed the experiment only once and had a potential performance of 100% (correctly reported both digits), 50%, or 0%. Note that participants did show better-than-chance performance on both the in-flight experiment and ground-based control (chance = 10% accuracy) even though the alternation period had been set to 6 ms below their threshold. This performance gain might be attributable to perceptual learning; it may also be because movement of the chronometer makes it slightly easier to read due to separation of successive frames, and participants sometimes moved the device involuntarily as they hit the net. To ensure parity between the comparisons, we applied a small jerk to control participants' wrists to mimic how the device moved when free-fall participants hit the net. Asterisks represent p<0.05.

Using the retrospective stopwatch estimation, participants' duration estimates increased by an average of 36% when they recalled their own fall (from 2.17±0.24 s to 2.96±0.51 s, p<0.03, paired t-test, one-tailed, Fig. 2a), consistent with their verbal report that their fall had “seemed to take a very long time”.

But was there a concomitant increase in temporal resolution? For a flickering display such as the one in this experiment, participants typically exhibit a sharp threshold between frame rates at which they can perceive the stimulus, and frame rates at which they cannot (around 3 ms, determined in a separate experiment). Therefore, we would expect that when measuring near their baseline threshold, a small increase in a participants' temporal resolution would translate into a large increase in perceptual performance. By fitting a psychometric curve to participants' pre-jump data, and then shifting it by the amount predicted by a 36% speed-up of their temporal resolution, we obtained an estimate of the perceptual performance we might expect during the free fall. The mean expected performance (measured as correct digit identification) is plotted in Figure 2b, along with the actual performance. In a control experiment, we repeated the experimental procedure, but participants remained on the ground. The mean in-flight performance was much less than what we predicted, and no different from the ground-based control (p = 0.98). Thus, while duration estimates increase during a high-fear situation (Fig. 2a), the lack of a matching increase in perceptual performance (Fig. 2b) indicates that duration estimates are not directly linked to temporal resolution.

## Discussion

We have tested whether the subjective duration increase of a frightening event is due to increased temporal resolution (as from the speeding of a camera) or instead whether duration distortions do not necessarily entail the expected consequences of a unitary time slowing down. Understanding how and why duration estimations change constrains neural models of time representation, some of which are turning to the question of whether the representation of time can speed or slow [1], [11], [12].

Participants free-falling from 50 meters experienced a duration expansion, yet they did not gain increased discrimination capacities in the time domain. This finding supports the hypothesis that subjective time is not unitary, and is consistent with the finding that flicker rates and the pitches of sounds do not lower during subjective duration dilations [13], as would be expected during slow motion in movies.

A critical point for the logic of this study is that flicker fusion frequencies are not limited by the retina, since retinal ganglion cells have extremely high temporal resolution. For example, in cat retinal ganglion cells, temporal resolution is ∼95 Hz for X cells and ∼120 Hz for Y cells under photopic illumination [14]. In primates, neurons in temporal cortex are able to temporally follow complex stimuli presented at 72 Hz [15]. Additionally, given the effect of many medications on the psychophysical flicker fusion frequency [16], it is clear that the limits of temporal resolution are central, not peripheral.

Therefore, at this stage there is no evidence to support the hypothesis that subjective time as a whole runs in slow motion during frightening events. Rather, we speculate that the involvement of the amygdala in emotional memory may lead to dilated duration judgments retrospectively, due to a richer, and perhaps secondary encoding of the memories [17]–[20]. Upon later readout, such highly salient events may be erroneously interpreted to have spanned a greater period of time.
